# Comparison of the trabecular meshwork length between open and closed angle with evaluation of the scleral spur location

**DOI:** 10.1038/s41598-019-43315-2

**Published:** 2019-05-02

**Authors:** Wungrak Choi, Min woo Lee, Hyun Goo Kang, Hye Sun Lee, Hyoung Won Bae, Chan Yun Kim, Gong Je Seong

**Affiliations:** 1 Department of Ophthalmology, Institute of Vision Research, Yonsei University College of Medicine, Seoul, Korea; 2 Biostatistics Collaboration Unit, Yonsei University College of Medicine, Seoul, Korea

**Keywords:** Glaucoma, Vision disorders

## Abstract

This study analysed the trabecular meshwork (TM) length in open and closed angle patients and presented a better method for locating the scleral spur using Schwalbe’s line method in anterior segment optical coherence tomography (AS-OCT). Patients who underwent AS-OCT at Yonsei University Gangnam Severance Hospital we enrolled; 58 and 57 open and closed angle eyes, respectively. We measured the distance from the scleral spur to Schwalbe’s line as the TM length and compared it between open and closed angle patients, and to previous studies. TM length was applied to locate the scleral spur using Schwalbe’s line method. Mean TM lengths were 811 ± 83 μm and 575 ± 96 μm in the open and closed angle groups, respectively (p < 0.001). Comparing the actual TM heights using Schwalbe’s line method with an updated reference distance significantly increased accuracy to locate the scleral spur (open: 811 μm, closed: 575 μm) when compared with the 1000 μm reference distance (p < 0.001). TM length was significantly different between open and closed angle patients. Further, the reference distance for Schwalbe’s line method should be distinguished according to open and closed angle eyes in order that the scleral spur can be located properly.

## Introduction

The position of the scleral spur is important as a reference point for identifying the anatomical structures of the anterior chamber of the eye. The scleral spur is located at the junction of the ciliary muscle fibres and anchoring region of the base of the trabecular meshwork (TM)^1^. Visualizing the scleral spur using anterior segment optical coherence tomography (AS-OCT) is important to determine if the anterior chamber angle is occluded^2^. Additionally, the anterior chamber angle width is measured in reference to the location of the scleral spur, and angle measurement is not possible if the scleral spur is obscured^2,3^. However, the scleral spur can be difficult to visualize in some AS-OCT images^3–5^. Furthermore, closed angle patients are more likely to have an undetectable scleral spur, although the exact relationship between scleral spur visibility and angle configuration remains unclear^5^. Various studies have sought to define the exact location of the scleral spur using AS-OCT. Seager *et al*. have introduced several methods to determine scleral spur location, including Schwalbe’s line method, which provides valuable evidence for the determination of the correct location of the scleral spur^6^. However, this study was conducted primarily in patients of Western descent.

Several previous studies have measured TM length with AS-OCT, but the results differed with regard to angle configuration, study region, and ethnicity^7–9^. TM anatomy plays an important role in aqueous humour outflow and intraocular pressure (IOP) elevation; therefore, its anatomical size may provide insight regarding the potential risk of glaucoma development and/or progression^7^.

It is important to study TM length and the applicable scleral spur detection methods in Asian populations, particularly Korean populations (in which TM length has never before been studied). The purpose of our study was to analyse the TM length of open and closed angle patients and present a better method to define the exact location of the scleral spur using Schwalbe’s line method in Korean patients. In this study, we hypothesized that there may be differences in the TM size according to the angle conformation, region, or ethnicity of the patients. Differences in TM size would indicate that different parameters should be used to assess the correct scleral spur location according to patient demographics.

## Results

To compare TM height, 115 eyes in 60 patients were enrolled in the study. Three eyes were excluded due to poor image quality (two open angle and one closed angle) and two fellow open angle eyes in the closed angle group were excluded. Therefore, 58 and 57 eyes were included in the open and closed angle group, respectively. The baseline characteristics are summarized in Table 1. The mean age of the patients was 64.6 and 49.6 years in the closed and open angle groups, respectively. The mean baseline IOP was 15.36 ± 5.54 and 13.29 ± 3.89 mmHg in the closed and open angle groups, respectively. The mean anterior chamber depth was 1.87 ± 0.35 and 2.99 ± 0.35 mm in the closed and open angle group, respectively (p = 0.019). The mean central corneal thickness (CCT) was 543.1 μm and 515.0 μm in the closed and open angle groups, respectively (p < 0.001). There was no significant difference in mean deviation (MD), visual Field Index (VFI), and mean retinal nerve fibre layer (RNFL) thickness between groups (p = 0.251, 0.928, and 0.243, respectively).

**Table 1:** Baseline clinical characteristics.

Characteristic	Closed angle eyes	Open angle eyes	P-value
**Age [years]**			
Mean ± SD	64.6 ± 10.7	49.6 ± 17.2	<0.001
Sex [eye]			0.088
Male	20 (35.1%)	29 (50.0%)	
Female	37 (64.9%)	29 (50.0%)	
Mean IOP ± SD (mmHg)	15.36 ± 5.54	13.29 ± 3.89	0.022
Spherical equivalent ± SD (D)	1.38 ± 3.67	−1.78 ± 2.95	<0.001
Anterior chamber depth ± SD (mm)	1.87 ± 0.35	2.99 ± 0.35	0.019
CCT ± SD (mm)	543.1 ± 35.3	515.0 ± 82.3	<0.001
MD (dB)	−3.61 ± 4.42	−2.47 ± 4.67	0.251
VFI (%)	93.30 ± 10.05	93.09 ± 10.97	0.928
Mean RNFL thickness (μm)	88.39 ± 14.48	84.91 ± 10.66	0.243

IOP: Intraocular pressure; SD: standard deviation; CCT: Central corneal thickness; MD: Mean deviation; VFI: Visual Field Index; RNFL: Retinal nerve fibre layer.

Independent two sample t-test and chi-square test was done.

### Visibility of the scleral spur and Schwalbe’s line

**Figure 1:**
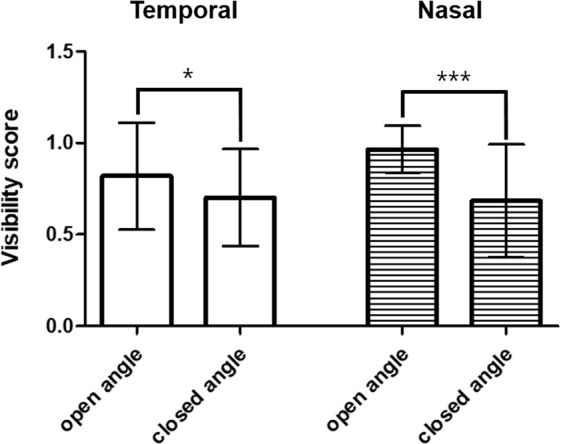
Visibility of the scleral spur and Schwalbe’s line. The visibility score between open and closed angle differed significantly in both the temporal (p = 0.026) and nasal (p < 0.001) positions. (*p < 0.05; ***p < 0.001).

Visibility of the scleral spur and Schwalbe’s line on the AS-OCT images was as follows: open angle (temporal: 95%, nasal: 98%) and closed angle (temporal: 98%, nasal: 93%). The mean (SD) visibility score was 0.82 (0.29) and 0.97 (0.13) for the temporal and nasal positions, respectively, in the open angle group, and 0.70 (0.26) and 0.68 (0.31) for the temporal and nasal positions, respectively, in the closed angle group. The visibility score between open and closed angles differed significantly both in the temporal (p = 0.026) and nasal (p < 0.001) positions (Fig. 1).

### Trabecular meshwork length (n = 115)

**Figure 2:**
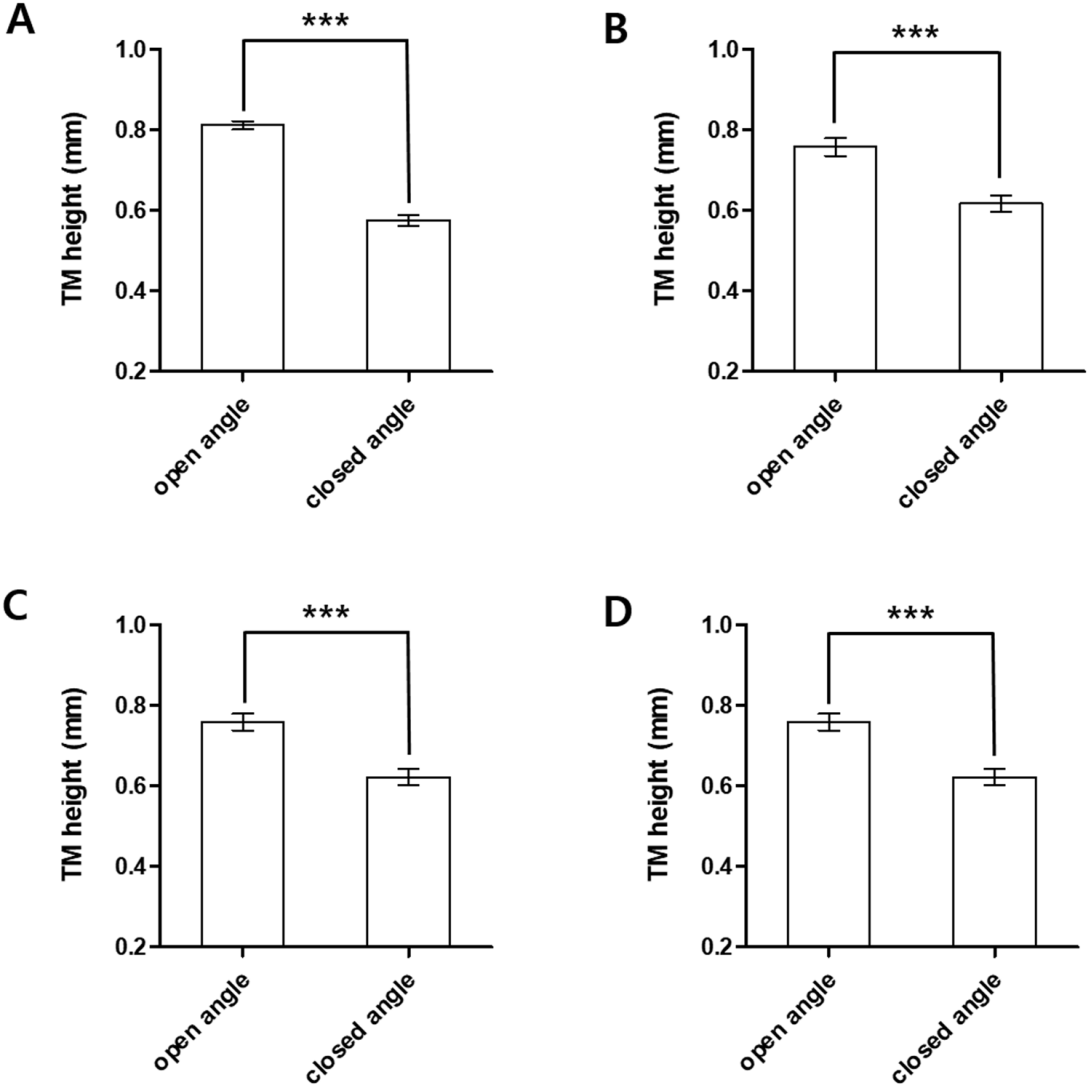
Trabecular meshwork length in open and closed angle eyes. The mean (SD) TM length was measured and was compared between open and closed angle patients (***p < 0.001). (**A**) Univariable; (**B**) Multivariable 1: result of adjusting all variables (univariable result p < 0.05 + clinically important variable); (**C**) Multivariable 2: corrected by subtracting the IOP that did not affect the TM length (univariable result, p < 0.05); (**D**) Multivariable 3: corrected by variables that affected TM height. A stepwise method was used to select only variables that were significant. IOP; intraocular pressure; TM: trabecular meshwork.

The mean TM length was 696 ± 148 μm. The open and closed angle mean TM lengths were 811 ± 83 and 575 ± 96 μm, respectively (Table 2). Age, baseline IOP, anterior chamber depth, spherical equivalent, and CCT were significantly different between the open and closed angle groups; therefore, multivariate analysis was performed to calibrate these demographics (Table 3). Both the univariable and multivariable analyses showed that mean TM length was significantly greater in open angle eyes than closed angle eyes (p < 0.001; Table 2 and Fig. 2).

**Table 2:** Trabecular meshwork length in open and closed angle eyes.

	Univariable		Multivariable 1		Multivariable 2		Multivariable 3	
Group	Mean TM height	SE	Mean TM height	SE	Mean TM height	SE	Mean TM height	SE
Closed angle eyes	575	13	617	20	622	20	622	20
Open angle eyes	811	11	757	22	758	21	758	21

Univariable and multivariable linear regression was performed.

Multivariable 1 is the result of adjusting all variables (univariable result p < 0.05 + clinically important variable).

Multivariable 2 was corrected by subtracting the IOP that did not affect the TM length (univariable result p < 0.05).

Multivariable 3 was corrected by variables that affected TM height, and a stepwise method was used to select only the variables that were significant.

SE: standard error; TM: trabecular meshwork.

**Table 3:** Linear regression of trabecular meshwork length and patient demographics.

	Univariable		Multivariable 1		Multivariable 2		Multivariable 3	
	B(SE)	p-value	B(SE)	p-value	B(SE)	p-value	B(SE)	p-value
**group**								
Closed angle eyes	Ref(0)		Ref(0)		Ref(0)		Ref(0)	
Open angle eyes	0.236(0.017)	<0.0001	0.236(0.017)	<0.0001	0.136(0.037)	0.0004	0.136(0.036)	0.0003
**Variable**								
age	−0.004(0.001)	<0.0001	−0.004(0.001)	<0.0001	−0.001(0.001)	0.4635		
**sex**								
Male	Ref(0)		Ref(0)		Ref(0)			
Female	−0.063(0.028)	0.0264	−0.063(0.028)	0.0264	0.001(0.020)	0.9558		
IOP	−0.004(0.003)	0.1325	−0.004(0.003)	0.1325				
Spherical equivalent	−0.019(0.003)	<0.0001	−0.019(0.003)	<0.0001	−0.008(0.003)	0.0058	−0.008(0.003)	0.0033
Anterior chamber depth	0.168(0.015)	<0.0001	0.168(0.015)	<0.0001	0.058(0.027)	0.0373	0.064(0.025)	0.0129
CCT	−0.001(0.001)	0.013	−0.001(0.000)	0.013	−0.001(0.001)	0.6263		

Univariable and multivariable linear regression was performed.

Multivariable 1 is the result of adjusting all variables (Univariable result, p < 0.05 + clinically important variable).

Multivariable 2 was corrected by subtracting the IOP that did not affect the TM length (Univariable result, p < 0.05).

Multivariable 3 was corrected by variables that affected TM height, and a stepwise method was used to select only the variables that were significant.

IOP: Intraocular pressure; CCT: Central corneal thickness; SE: standard error.

To confirm our results, a sensitivity test was performed by randomly selecting one eye from each individual and re-analysed the data to see if the same results were recorded. The sensitivity test also revealed a significant difference in mean TM height between the open and closed eyes (p < 0.001; Supplemental Data 1).

In addition, to investigate the difference of TM length by angle closure type, we divided the closed angle group into subgroups: primary angle closure suspected (PACS), primary angle closure (PAC), or primary angle-closure glaucoma (PACG). Interestingly, there was no significant difference in TM length between angle closure subgroups (p = 0.336; Supplemental Data 2).

### Application of the reference distance (n = 60)

**Figure 3:**
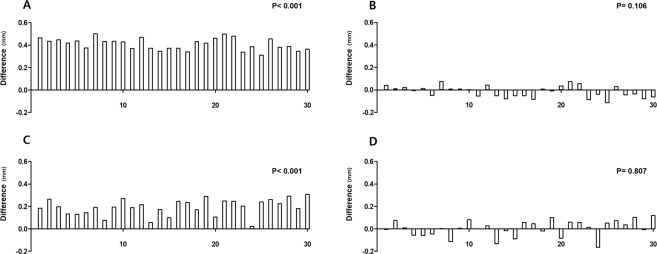
Application of the reference distance to the trabecular meshwork. The reference distance was applied and was compared to the actual TM length. (**A**) Difference between the actual TM length and the reference distance (1 mm) in closed angle eyes (p < 0.001). (**B**) Difference between the actual TM length and the reference distance (mean TM length: 0.575 mm) in closed angle eyes (p = 0.106). (**C**) Difference between the actual TM length and the reference distance (1 mm) in open angle eyes (p < 0.001). (**D**) Difference between the actual TM length and the reference distance (mean TM height: 0.811 mm) in open angle eyes (p = 0.807); TM: trabecular meshwork.

The TM reference distance was randomly applied to patients for comparison with Schwalbe’s line method. First, reference distances of 811 μm and 575 μm for the open and closed angle, respectively, were applied. Second, the reference distance of 1000 μm (1 mm), which was recommended in a previous study, was applied^6^. When we compared the second reference distance (1000 μm) to the TM height, there was a significant difference between the actual TM length and reference distance. However, the difference between the actual TM length and first reference distances was not significant (Fig. 3).

## Discussion

In this study, we sought to analyse TM length in open and closed angle patients, and report a better method for locating the scleral spur using Schwalbe’s line method *via* AS-OCT. We reported increased accuracy for locating the scleral spur using actual TM length, measured using Schwalbe’s line method with an updated reference distance, when compared with using the 1000 μm reference distances. In addition, TM length was significantly different between open and closed angle patients.

Currently, AS-OCT is one of the most important imaging modalities for anterior segment evaluation^10^. In AS-OCT imaging, the position of the scleral spur is an important reference point for identifying the anatomical structures of the anterior chamber. Seager *et al*. have claimed that using the distance from Schwalbe’s line to the scleral spur can be a convenient method to determine the scleral spur location when it is difficult to detect^6^. The distance from Schwalbe’s line to the scleral spur represents the TM height; therefore, this method could provide valuable data to determine the correct location of the scleral spur. The authors of this previous study used the reference distance (TM height) of 1000 μm (1 mm) to locate the scleral spur and claimed the results were accurate^6^. However, they did not separate the reference distances between open and closed angles when presenting their results.

Although we excluded poor images that did not allow for any distinction of the scleral spur and Schwalbe’s line, the visibility score remained significantly lower in closed angle patients than open angle patients. This may indicate that patients with closed angle eyes are more likely to have an undetectable scleral spur; therefore, the use of Schwalbe’s line method may be more effective in closed angle patients. Accordingly, it is necessary to verifying applicable methods in open and closed angle eyes.

The mean TM length in closed angle patients was 575 μm in our study, which was significantly different from the open angle mean TM height and the previously claimed reference distance (1000 μm)^6^. Additionally, the data from our study showed a similar trend with the open angle patients. For both open and closed angle patients, the difference between the actual TM length and the reference distance was significantly greater than the previously claimed reference distance, particularly when compared with the mean TM length that was described in our study. These results indicate that the previously claimed reference distance should be distinguished by angle configuration, and that Schwalbe’s line method was more accurate when using these updated reference distances.

Our study yielded relatively different results compared with previous studies. Chen *et al*. have compared TM length by ethnicity in open angle patients in America^7^, and found mean TM heights as follows: white (851 μm), Asian (843 μm), Hispanic (822 μm), and African American (771 μm)^7^. However, Tun *et al*. have reported that the TM length in Chinese patients is: temporal 724 μm (open angle: 734 μm, closed angle: 696 μm) and nasal 712 μm (open angle: 717 μm, closed angle: 698 μm)^9^. Although the majority of Asians were Chinese in both studies, the results are noticeably different. We showed greater open angle TM length in Koreans than “Singapore Chinese”; however, it was less than “American Chinese”. The reason for the different results in Asian populations should be studied in the future. This difference may have been caused by geographical factors, ethnicity, and other genetic factors.

Cheung *et al*. have reported no significant difference in TM length between open and closed angle eyes in Singapore^11^. Masis *et al*. have reported complementary results to our study; a significant difference between open and closed angle TM length^8^. The reason for these contrasting results might be due to differences in sample size or ethnicity; we recruited Korean patients alone, whereas the majority of the study population was Chinese in the Singapore study. Combining these results, although they are broadly tied to Asian populations, genetic and anatomical factors may differ depending on the region and country. Further studies should assess these differences.

Interestingly, the difference in TM length was much larger than reported in previous studies^8^. This might be due to the different definitions of angles in the recruited patients in our study. The definition of a closed angle was >270° of a posterior TM invisible in our study, when compared with 180° in the previous study. Additionally, the type distribution of the closed angle may have affected the results. More than half of the closed angle patients were PACG in our study. Although this was not significant, there was a trend for a shorter TM length in PACG than PACS, which may have also affected the result. In addition, selection bias may have been a factor, because recruited patient numbers were not large in both studies.

Further, the mean age in the closed angle group was significantly higher than in the open angle group. Age was a significant factor that affected TM length. The result is most likely to be affected due to the 15-year gap between compared groups. Therefore, we performed a multivariate analysis to distinguish these various factors and found that the actual TM length difference was approximately 130 μm, not 250 μm, which was comparable to a previous study^8^.

There may be reasons underlying the short TM length in patients with closed angle eyes. Patients with a shorter TM length may transform more easily to a closed angle during aging. The risk of cataracts development increases with age, and may increase the volume of the lens. This pushes the iris towards the peripheral cornea and eventually results in age-related anterior chamber narrowing and angle closure. Therefore, patients with a shorter TM length would be more likely to develop to a closed angle.

Furthermore, the extracellular matrix increases and cellularity in the TM decreases with age, which results in a reduced outflow facility of the eye^12–14^. Therefore, a shorter TM may be more sensitive to the effects of aging on these physiological changes. This may explain the fact that aging is related to angle closure glaucoma^15–17^.

Alternatively, the closed angle shows more anterior rotation of the ciliary process because the lenses are more forward-shifted. This situation would lead to less ciliary muscle tension on the scleral spur, leading to less “stretching” of the TM, which results in a shorter TM length^8^. In summary, shorter TM length may be associated with the pathophysiology of angle closure, and our findings may provide insight into the mechanisms underlying angle closure.

The limitations of our study include the retrospective design and relatively small sample size. In addition, the study was conducted in Korean patients alone, and not all of the participants were patients with glaucoma. However, our data clearly demonstrated a difference between open and closed angle TM height. Future studies with larger sample sizes should be conducted to confirm our results. Another limitation is that due to the retrospective study design, authors were not able to correlate TM and axial lengths. We performed regression analysis using anterior chamber depth, which is known to be, at least partially, associated with axial length^18,19^. We showed that anterior chamber depth was associated with TM length in both univariable and multivariable regression analysis. Although anterior chamber depth is not only related to axial length, there may be possibility that axial length may be related to TM length. Further, we did not measure TM length in the superior and inferior positions. Limitation of scleral spur visibility is more common in the superior and inferior positions than in the nasal and temporal positions; therefore, this should also be studied in the future^20^.

In conclusion, TM length was significantly different between open and closed angle patients. Therefore, the reference distance for Schwalbe’s line method should be distinguished according to patients with open and closed angle when it is being used to locate the scleral spur.

## Methods

### Patient enrolment

This was a retrospective, observational, single-centre study. Of the patients who were imaged with AS-OCT at Yonsei University Gangnam Severance Hospital between January 2015 and December 2017, 30 patients with open angles and 30 patients with closed angles were enrolled in the study.

First, patients imaged with AS-OCT were divided into open or closed angle group according to their diagnosis. Next, 30 patients were randomly selected from open angle group and 30 patients were randomly selected from closed angle group.

A systemic evaluation of their medical records was performed and the complete ocular examination results were reviewed. Ocular examinations included current ophthalmologic diagnosis, visual acuity measurement, IOP measurement, review of the gonioscope records, spherical equivalent measurement, CCT measurement, anterior chamber depth measurement, MD and VFI of visual field test, mean RNFL thickness, and TM length measurement. To avoid the drawback of poor image quality in closed angle eyes, the inclusion criteria included the necessity to distinguish the scleral spur and Schwalbe’s line in at least one eye AS-OCT image in randomly selected patients. The exclusion criteria were history of previous intraocular surgery, penetrating trauma, and cornea opacities that can disrupt AS-OCT imaging. Additionally, if the patient had a closed angle in one eye and an open angle in the other, the other eye was excluded from the study depending on which group the patient was categorized.

Approval for this study was obtained from the Gangnam Severance Hospital Institutional Review Board, which provided a waiver of informed consent for the retrospective review of existing patient records (IRB number: 3-2018-0052). The methods adhered to the tenets of the Declaration of Helsinki and were HIPAA compliant.

### Study design

Sixty randomly enrolled patients (120 eyes) who underwent AS-OCT were retrospectively reviewed. An occludable angle was defined as an angle with <90° of posterior TM visible^21^. We distinguished subgroups within the closed angle group as PACS, PAC, and PACG, based on recommendations from the International Society for Geographical &Epidemiological Ophthalmology (ISGEO). Briefly, PACS was defined as an eye with an occludable angle without peripheral anterior synechiae or glaucomatous change of the optic disc/visual field. PAC was defined as an eye with any degree of peripheral anterior synechiae or with an occludable angle accompanied by an elevated IOP (21 mm Hg) and/or iris ischemia but without glaucomatous damage on the optic disc/visual field test^17,21^. PACG was defined as an eye with glaucomatous damage to the optic nerve/visual field in the presence of PAC^17,21^.

TM length was measured in the nasal and temporal side of the eye using AS-OCT. We used one image along the horizontal meridian which was taken in dark light condition. We manually selected the 3 and 9 o’clock positions as the nasal and temporal positions. The mean TM length (nasal and temporal) was compared between open and closed angle patients.

### AS-OCT measurements

Anterior segment images were obtained using swept source optical coherence tomography with CASIA AS-OCT (CASIA SS-1000, Tomey Corporation, Nagoya, Aichi, Japan). Independent observers performed all measurements. We used normal-resolution scan mode for the results and used the recently described band of extracanalicular limbal lamina (BELL) method to measure the TM length^22^. The visibility of the scleral spur and Schwalbe’s line was checked to exclude poor quality images and the visibility score was measured (0: poor visibility, 0.5: moderate visibility, 1: good visibility).

**Figure 4:**
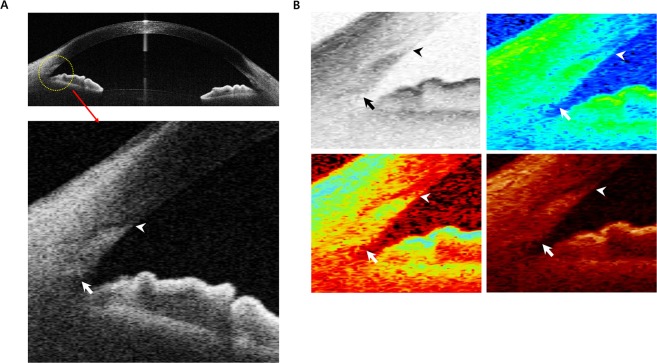
AS-OCT measurement of the trabecular meshwork length. TM length was measured using the distance between the scleral spur (arrow) and Schwalbe’s line (arrow head). (**A**) Image of the anterior chamber angle taken by AS-OCT. (**B**) Various image modes in the AS-OCT were referred to properly measure the TM height. From the left to the right, the image versions are invert grey mode, rainbow mode, gradient mode, and temperature mode. AS-OCT: anterior segment optical coherence tomography; TM: trabecular meshwork.

TM length was measured using the distance between the scleral spur and Schwalbe’s line. Scleral spur was identified with the bump seen as an internal projection of sclera into the anterior chamber or following the interface between the sclera and the ciliary muscle until it intersects a line projected along the inner cornea^6^. To locate the Schwalbe’s line more accurately, we used the BELL method. BELL was defined as a hyporeflective band wrapping around the TM and Schlemm’s canal^22^. Location of Schwalbe’s line was defined as corneal termination of a hypo-reflective band^22^. The location was confirmed using the tip of the U-shaped interface between the cornea and the sclera^6,9^. Schwalbe’s line was assumed to be at the inner apex of the U-shape interface. Additionally, as CASIA AS-OCT has various modes of image conversion, the included software tools were utilized to properly measure the TM length (Fig. 4).

### Application of the reference distance to the TM

The accuracy of Schwalbe’s line method was compared according to which reference distance was used. The two reference distances of TM length were compared to the actual TM length of patients who had already undergone AS-OCT examination. Sixty newly enrolled eyes (30 closed angle eyes and 30 open angle eyes) were randomly selected to be compared using two reference distances. The first reference distance was the mean TM length of open and closed angles, and the second reference distance was 1 mm (1000 μm), which has been reported previously^6^.

### Statistics

Data were analysed using SPSS 22.0 software (SPSS, Chicago, IL, USA) and SAS (version 9.3, SAS Inc., Cary, NC, USA). Differences between groups were examined using the independent t-test, independent two sample t-test, one-way ANOVA test, and chi-square test. P < 0.05 was considered statistically significant. The relationship between TM length and angle configuration (open and closed angle) was analysed using univariable and multivariable linear regression analyses.

Multivariable analyses were confirmed using 3 different methods. First, we adjusted for the important clinical variables that had been measured. Second, we subtracted factors that did not affect TM length (univariable result, p > 0.05). Third, a stepwise method was used to select only variables that were significant, and selected significant factors were adjusted for analysis.

To confirm our result, we performed a sensitivity test by randomly selecting one eye of each individual and re-analysing the data to verify the same results.

## Supplementary information

Supplementary Dataset 1

## Data Availability

The datasets used and/or analysed during the current study are available from the corresponding author on reasonable request.
