# Supplementary material for: Comparison of the trabecular meshwork length between open and closed angle with evaluation of the scleral spur location
Source: Sci Rep. 2019 May 2;9:6857. doi: 10.1038/s41598-019-43315-2 (PMC6497692; doi:10.1038/s41598-019-43315-2)
Supplement: Supplementary file 1 — Supplementary Dataset 1 [file 41598_2019_43315_MOESM1_ESM.pdf]

Comparison of the trabecular meshwork length between open and closed angle with evaluation of the scleral spur location

Wungrak Choi,[1] Min woo Lee,[1] Hyun Goo Kang,[1] Hye Sun Lee,[2] Hyoung Won Bae,[1] Chan Yun Kim,[1] Gong Je Seong[1]*

[1]Department of Ophthalmology, Institute of Vision Research, Yonsei University College of Medicine, Seoul, Korea

[2]Biostatistics Collaboration Unit, Yonsei University College of Medicine, Seoul, Korea

*Corresponding author: Gong Je Seong, MD

Department of Ophthalmology, Gangnam Severance Hospital, 211 Eonju-ro, Gangnam-gu, Seoul 06273, Korea.

E-mail: GJSEONG@yuhs.ac

Phone: 82-2-2019-3440

Fax: 82-2-3463-1049

Supplemental data 1: Sensitivity test

A.

**Characteristic**	**Closed angle eyes (n = 29)**	**Open angle eyes (n = 30)**	**P-value**
Age [years]			
Mean ± SD	64.6 ± 10.8	49.6 ± 17.4	<0.0001
Sex [eye]			0.088
Male	10 (34.5%)	15 (50.0%)	
Female	19 (65.5%)	15 (50.0%)	
Mean IOP ± SD (mmHg)	15.38 ± 6.30	13.21 ± 3.80	0.1189
Spherical equivalent ± SD (D)	1.28 ± 3.80	–1.69 ± 2.81	0.0029
Anterior chamber depth ± SD (mm)	1.87 ± 0.38	2.99 ± 0.34	<0.0001
CCT ± SD (mm)	543.4 ± 34.4	509.6 ± 108.0	0.1185
Mean TM height(μm)	585 ± 101	809 ± 88	<0.0001

B.

	**Univariable**		**Multivariable 1**		**Multivariable 2**		**Multivariable 3**	
**group**	**Mean TM length**	**SE**	**Mean TM length**	**SE**	**Mean TM length**	**SE**	**Mean TM length**	**SE**
**Closed angle eyes**	585	19	615	33	626	32	603	19
**Open angle eyes**	809	16	770	37	765	34	791	20

The sensitivity test was performed by randomly selecting one eye of each individual and data were re-analyzed to confirm the results

A. Demographics of the sensitivity test, independent two sample t-test, and chi-square test that were performed.

B. Univariable and multivariable linear regression was performed. Multivariable 1 is the result of adjusting all variables (univariable result, $p < 0.05$ + clinically important variable). Multivariable 2 was corrected by subtracting the IOP that did not affect the TM length (univariable result, $p < 0.05$). Multivariable 3 was corrected by variables that affected TM length, and a stepwise method was used to select only the variables that were significant.

IOP: Intraocular pressure; SD: Standard deviation; CCT: Central corneal thickness; SE: standard error; TM: trabecular meshwork.

Supplemental data 2: Trabecular meshwork length in closed angle eyes

Sub group	PACS		PAC		PACG		
Value	Mean TM length	SE	Mean TM length	SE	Mean TM length	SE	P-value
	603	22	555	34	567	60	0.336

One-way ANOVA was performed.
PACS: Primary angle closure suspect; PAC: Primary angle closure; PACG: Primary angle closure glaucoma; SE: standard error; TM: trabecular meshwork
